# Preparing medical students for clinical practice: easing the transition

**DOI:** 10.1007/s40037-017-0352-2

**Published:** 2017-04-10

**Authors:** Alexandra R. Teagle, Maria George, Nicola Gainsborough, Inam Haq, Michael Okorie

**Affiliations:** 1 Department of Medicine, Brighton and Sussex University Hospitals NHS Trust, Eastern Road, Brighton, UK; 2 Brighton and Sussex Medical School, Brighton, UK

**Keywords:** Medical students, Clinical practice, Preparation for practice, Simulation

## Abstract

The transition from medical student to junior doctor is a challenge; the UK General Medical Council has issued guidance emphasizing the importance of adequate preparation of medical students for clinical practice. This study aimed to determine whether a junior doctor-led simulation-based course is an effective way of preparing final year medical students for practice as a junior doctor.

We piloted a new ‘preparation for practice’ course for final year medical students prior to beginning as Foundation Year 1 (first year of practice) doctors. The course ran over three days and consisted of four simulated stations: ward round, prescribing, handover, and lessons learnt. Quantitative and qualitative feedback was obtained.

A total of 120 students attended (40 on each day) and feedback was collected from 95 of them. Using a scale of 1 (lowest) to 5 (highest), feedback was positive, with 99% and 96% rating 4 or 5 for the overall quality of the program and the relevance of the program content, respectively. A score of 5 was awarded by 67% of students for the ward round station; 58% for the handover station; 71% for the prescribing station, and 35% for the lessons learnt station. Following the prescribing station, students reported increased confidence in their prescribing.

Preparation for practice courses and simulation are an effective and enjoyable way of easing the transition from medical student to junior doctor. Together with ‘on-the-job’ shadowing time, such programs can be used to improve students’ confidence, competence, and ultimately patient safety and quality of care.

**Electronic supplementary material:**

The online version of this article (doi: 10.1007/s40037-017-0352-2) contains supplementary material, which is available to authorized users.

## Introduction

The transition from medical student to junior doctor – from supported learning to independent practice – is a challenge. In recognition of this, the UK doctors’ statutory regulator, the General Medical Council (GMC), produced a document entitled *Tomorrow’s Doctors *(now referred to as* Promoting excellence: standards for medical education and training)* [1], emphasizing the importance of preparing students for the first year of clinical practice as a doctor, known as Foundation Year 1 (F1). However, a large proportion of UK medical graduates admit to feeling unprepared. This was highlighted over 10 years ago by Goldacre et al., who found that over 40% of UK medical graduates felt under-prepared for practice as a doctor [2]. Furthermore, while graduates may feel prepared for basic clinical tasks such as history taking and examination, a lack of exposure to clinical situations in medical school leaves them unprepared for more complex tasks such as management of acutely unwell patients, prescribing, managing workload and on-call duties [3].

Evidence suggests that mortality increases during each August [4], the time when new F1 doctors begin work, as a result of lack of experience, with the first day of work for brand new F1s colloquially referred to as ‘Black Wednesday’. In light of this, many National Health Service foundation schools now offer a period of induction and/or shadowing preceding full commencement of independent practice, in line with GMC guidance [1]. Prescribing errors, the area of clinical practice new F1 doctors are most concerned about [3], can lead to serious or even fatal consequences: the EQUIP study found errors in 9% of hospital prescriptions [5]. Subsequently, the national Prescribing Safety Assessment was developed by the British Pharmacological Society and Medical Schools Council, and rolled out in UK medical schools in 2014.

In response to these issues, we piloted a new ‘Preparation for Practice’ course, using simulation and real-life scenarios to cover common challenges for F1 doctors. This was driven by student feedback highlighting inadequate attention to the practical aspects of the transition from student to doctor. The purpose was to determine whether such an approach could improve participants’ knowledge and ability to manage the situations used, and better prepare them for clinical practice.

## Methods

### Course design and delivery

A total of 120 final year medical students from the Brighton and Sussex Medical School, UK (BSMS) 5‑year medical undergraduate degree attended the course over three days (40 on each day). The course was obligatory and was placed after final examinations so that students were more motivated and engaged than earlier in the curriculum, and were about to graduate and commence working as F1 doctors.

The course utilized simulation and comprised four stations: ward round, prescribing, handover and ‘lessons learnt’. The content was defined using feedback from former BSMS students and focus groups with junior doctors, which had demonstrated the need for practical experience that was not easily accessible through shadowing. These four areas were identified as often experienced in the first weeks of an F1’s career.

The ward round station comprised a simulated ward round with a consultant and junior doctors. Students, in simulated roles as junior doctors, were tasked with presenting the history of the ‘patient’, while their colleague wrote the medical notes and assessed the patient’s charts, all in real-time, including interruptions that the participants were required to manage. The roles were then switched, with one student leading the ward round in the consultant’s position. The purpose of this was to highlight the importance of structure, preparation and organization of the ward round, as well as multi-tasking, communication and teamwork.

The practical prescribing session was led jointly by junior doctors and pharmacists. It consisted of four case-based scenarios – insulin, analgesia and opiates, fluids and blood products, antibiotics – through which students worked in groups of three, using resources such as the British National Formulary and local prescribing guidance. These topics were chosen as they reportedly cause confusion for junior doctors and are potentially dangerous if prescribed incorrectly.

In the handover station junior doctor facilitators simulated handing over a patient to the participants, whilst being continuously interrupted by colleagues, pagers and phone calls. Through this, students observed the importance of a clear handover of each patient, and the impact of interruptions on the effectiveness of handover and patient safety. Students were then given the history and tasks for another ‘patient’, and simulated handing over to their colleague using a structured Situation, Background, Assessment, Recommendation (SBAR) approach, which had been learnt earlier in the station.

Finally, a ‘lessons learnt’ station consolidated the day’s themes by using examples of incidents in which patients had been or could have been harmed due to clinical errors, and discussing how the system or culture could be changed to prevent further similar occurrences.

All teaching and simulation was done by Foundation Year 1 and 2 doctors and pharmacists, and each session was supervised by a Consultant. The stations were structured sequentially, simulating the course of events in clinical practice – decision (ward round), intervention (prescribing), continuity of care and monitoring (handover) and reflection on clinical practice (lessons learnt). Prior to the course, junior teachers were given informal training in delivery of the content and feedback. Feedback was given directly to the students at the end of each session.

### Course evaluation

Students completed a confidential questionnaire/feedback form after the course (see Online Supplementary Material). This asked them to rate the course program on a modified Likert scale, from 1 (lowest) to 5 (highest), in terms of quality of program, relevance of content, and schedule of activities. Students also rated each station using this scale. The questionnaire asked students to retrospectively rate their confidence about prescribing before and after the prescribing station, using the modified Likert scale, with 1 being not confident at all, and 5 being very confident. Finally, the questionnaire asked students for free text comments on the best aspects of the course, and areas for improvement. Participants were informed that their feedback might be used for publication purposes, and by completing the feedback form, their consent for this was assumed.

## Results

**Fig. 1:**
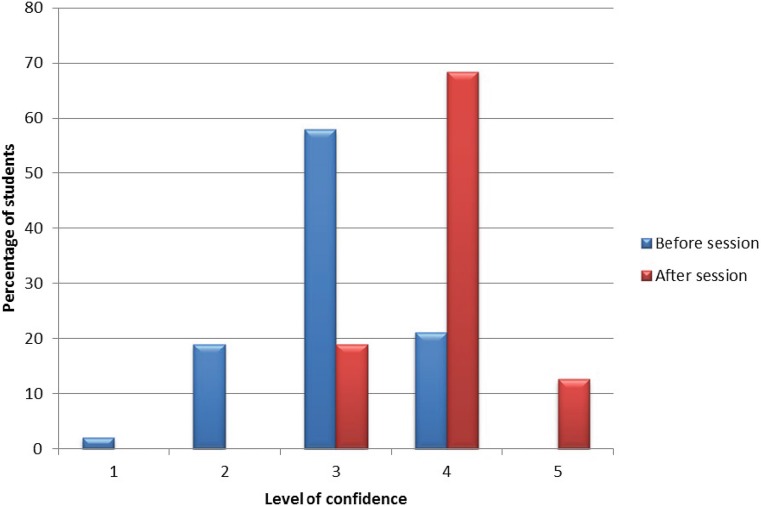
Students’ level of confidence with prescribing before and after the prescribing station; 5 = highest confidence and 1 = lowest confidence

Feedback was obtained from 95 of the 120 students: 94 students (99%) gave a rating of 4 or 5 for the overall quality of the program, 91 students (96%) rated 4 or 5 for the relevance of the program content, and the same results were obtained for the schedule of activities. A rating of 5 was awarded by 67% of students for the ward round station, 71% for the prescribing station, 58% for the handover station, and 35% for the lessons learnt station. Students reported increased confidence with prescribing compared with before the session, by at least one point on the scale, and overall confidence of the group was markedly improved (Fig. 1).

The qualitative feedback (free text comments) was overall very positive and was collated in the appropriate domains (general, ward round, prescribing, handover and lessons learnt) (Table 1). This was with the intention of building on and improving the course in the future.

**Table 1:** Qualitative feedback

Domain	Quotes from students
General	‘Totally relevant to being an F1!’ ‘Interactive, practical, and encouraging. It was Fantastic!’ ‘Thank you! The best prep for practice day we’ve had. It would be better to have more practical days like this and less lectures’ ‘Really useful picking on key topics – the only useful preparation for practice so far as it applies to everyone up and down the country’ ‘On the spot feedback was great’ ‘Having practical sessions covering what we have learnt in lectures, using practical skills helped cement the theory in my mind’ ‘Teaching like this much earlier and more frequently in medical school – much more relevant than much of what we learn’ ‘More sessions like this and less lectures please’ ‘Handover and Ward Round sessions were surprisingly useful’
Prescribing	‘Prescribing session was excellent’ ‘Having the pharmacists available was really helpful’ ‘Prescribing theory and actually writing prescription was really useful’ ‘It was extremely useful to learn how to prescribe insulin, analgesia, fluids, blood products and antibiotics’ ‘Insulin prescribing station was excellent’ ‘Having small groups in this session was helpful’ ‘This needed a longer session’ ‘More prescribing sessions, for example having a similar session before finals as practice makes perfect’
Ward rounds	‘Very insightful session’ ‘This session was excellent’ ‘The ward round feedback was great’ ‘A real eye opener’ ‘How to run a ward round was a very useful session. It was good to try to present a patient and keep records in real time’ ‘More of these sessions throughout training’ ‘More time on this session would be good so that everyone gets a chance to lead’
Handover	‘The handover session was invaluable’ ‘Handover was really helpful – hadn’t prioritized receiving information before’ ‘Practicing receiving handovers was a first and thoroughly useful’ ‘Practice handovers were very useful and constructive’ ‘More handover sessions please’
Lessons learnt	‘This session was really interesting’ ‘More cases in lessons learnt would be good’ ‘Could this session be shortened to allow for longer in the prescribing session’

## Conclusion

The importance of adequate preparation of medical students for clinical practice cannot be overemphasized [1]. However, this may be suboptimal [6], leaving medical graduates feeling unprepared [2, 3, 7, 8], particularly in more complex tasks such as acute care and prescribing [3, 7]. These concerns are shared by senior doctors [7, 9]. We have designed a simple course to expose graduating medical students to common scenarios faced by junior doctors. We believe that this course has helped improve the transition from medical student to junior doctor by using simulation, such that students were able to actively participate in realistic tasks and experience the scenarios in real-time. In addition, a near-peer teaching approach, with scenarios and workshops led by junior doctors and other junior members of staff, differentiated this from other learning environments such as lectures and consultant-led tutorials. Prescribing was a crucial component, as this is a challenging area for newly qualified doctors [3, 5], in which errors are potentially catastrophic [5]. This course was perceived to be an effective and enjoyable method of preparing for work as an F1 doctor and this was reflected in the positive feedback from final year medical students who participated. Combining this approach with ‘on-the-job’ shadowing time could potentially have far reaching positive effects on newly qualified doctors’ confidence and competence, and more importantly could have wider implications for safety and quality of patient care.

## Caption Electronic Supplementary Material

Confidential questionnaire/feedback form the students completed after the course.

## References

[1] General Medical Council (2009). Tomorrow’s doctors: outcomes and standards for undergraduate medical education.

[2] Goldacre M, Lambert I, Evans J, Turner G (2003). PRHOs’ views on whether their experience at medical school prepared them well for their jobs: national questionnaire survey. BMJ.

[3] Illing J, Morrow G, Kergon C (2008). How prepared are medical graduates to begin practice? A comparison of three diverse UK medical schools.

[4] Jen MH, Bottle A, Majeed A, Bell D, Aylin P (2009). Early in-hospital mortality following trainee doctors’ first day at work. PLOS ONE.

[5] Dornan T, Ashcroft D, Heathfield H (2009). An in depth investigation into cause of prescribing errors by foundation trainees in relation to their medical education. EQUIP study.

[6] Brown S, Bruce L, Clarke D (2015). General Medical Council, UK. The state of medical education and practice in the UK 2014.

[7] Tallentire VR, Smith SE, Wylde K, Cameron HS (2011). Are medical students ready to face the challenges of Foundation training?. Postgrad Med J.

[8] Evans DE, Roberts CM (2006). Preparation for practice: how can medical schools better prepare PRHOs?. Med Teach.

[9] Matheson C, Matheson D (2009). How well prepared are medical students for their first year as doctors? The views of consultants and specialist registrars in two teaching hospitals. Postgrad Med J.

