# Supplementary material for: Preparing medical students for clinical practice: easing the transition
Source: Perspect Med Educ. 2017 Apr 10;6(4):277–80. doi: 10.1007/s40037-017-0352-2 (PMC5542891; doi:10.1007/s40037-017-0352-2)
Supplement: Supplementary file 1 — Confidential questionnaire/feedback form the students completed after the course. [file 40037_2017_352_MOESM1_ESM.docx]

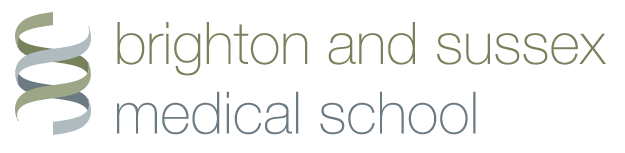


**Practical Preparation for Practice**

25th - 27th May 2014

We appreciate your help in evaluating this program. Please indicate your rating of the presentation in the categories below by circling the appropriate number, using a scale of 1 (low) to 5 (high).

1. How do you rate the practical preparation for practice day with respect to the issues listed

Overall quality of the program 1 2 3 4 5

Overall usefulness of the content provided 1 2 3 4 5

Overall organization and schedule of the program 1 2 3 4 5

2. What overall score would you give the sessions covered today?

How to run a ward round 1 2 3 4 5

Handover 1 2 3 4 5

Prescribing 1 2 3 4 5

Lessons Learnt 1 2 3 4 5

3. With regard to your prescribing skills, please indicant your level of confidence BEFORE the session:

1 2 3 4 5

Not Confident at all Low confidence Average Quite Confident Very Confident

Please indicate your level of confidence with regard to your prescribing skills AFTER today’s session:

1 2 3 4 5

Not Confident at all Low confidence Average Quite Confident Very Confident

4. Is there anything you especially liked about today’s event?

5. If these sessions were held again, what improvements or subjects would you like us to consider?

With thanks

Brighton and Sussex Medical School
